# The Effects of Emotion, Spokesperson Type, and Benefit Appeals on Persuasion in Health Advertisements: Evidence from Macao

**DOI:** 10.3390/bs13110917

**Published:** 2023-11-10

**Authors:** Ling Jiang, Huihui Liu, Nan Jiang

**Affiliations:** 1 School of Business, Macau University of Science and Technology, Macao 999078, China; lijiang@must.edu.mo (L.J.); 1909853gbm30009@student.must.edu.mo (H.L.); 2 College of Culture and Media, Guiyang University, Guiyang 550005, China

**Keywords:** pandemics, health communications, persuasiveness, spokesperson, emotional appeal, benefit appeal

## Abstract

Enhancing public awareness for epidemic prevention is crucial for safeguarding public health. This experimental study investigated the effectiveness of a combined approach involving three persuasive elements in public health advertising. Specifically, the study examined the interplay between emotional appeals (fear messages versus efficacy messages) and spokesperson type on the public’s response to health announcements. The results demonstrated that fear messages were more persuasive when conveyed by real human spokespersons (versus animated spokespersons), whereas efficacy messages were more acceptable when conveyed by animated spokespersons (versus real humans). Furthermore, the study revealed that the impact of emotional appeals and spokesperson type is moderated by benefit appeals (self-benefit or other-benefit). The joint effects of these persuasive variables on individuals’ intention to adopt preventive measures indicated that the interactions significantly differed across the two types of benefit appeal. Taken together, the findings represent a pioneering contribution to the field of health communication by comparing the persuasive effects of different combinations of emotional appeals, spokesperson types, and benefit appeals on public behavior. These findings offer practical guidance for public communicators to design more appropriate health advertisements based on the results of this study, thereby enhancing public acceptance of disease prevention measures.

## 1. Introduction

Due to the ongoing outbreaks of global pandemics, disease prevention has become an increasingly critical focus for health communicators. It is crucial to enhance public acceptance of specific epidemic prevention recommendations and encourage the adoption of personal health management measures, such as vaccination. How do different combinations of persuasive elements in health advertisements impact public acceptance? Are there variations in the persuasiveness of different tones and message types when it comes to motivating the public to take preventive measures? Although previous studies have explored the persuasive effects of health advertisements [1,2,3,4,5], none have empirically examined the impact of different combinations of emotional appeals, spokesperson types, and benefit appeals. To shed light on these matters and assist health communicators in achieving more effective communication outcomes, this study conducted an experimental study to examine the persuasive power of health advertisements featuring various combinations of these three elements on public acceptance and willingness to engage in preventive measures.

Consider a public health message for the prevention of a pandemic, such as the recent coronavirus, which states “Elderly people are more susceptible to infection, and they are also prone to severe illness or even death”. In contrast, consider another health message that states “The first confirmed patient recovered and was discharged from hospital, strengthening our confidence in fighting against the pandemic”. Both ads convey information about a pandemic prevention campaign, but the difference between the two is that one does so by evoking a negative emotion response (threat and fear), while the other arouses a positive emotion response (efficacy and hope) used to persuade the reader. Either of these persuasive techniques, which are extensively used in public health messages, can evoke negative or positive emotional responses [1,2,3,4,5].

Prior research on public health ads has mainly focused on the informational content of the message, whereas little attention has been given to the non-informational content of the message [6]. One of the significant persuasive visual tools has been the characters used as spokespersons in advertising [7]. In addition to using human spokespersons, communicators can also draw viewers’ attention to their ads through the use of animated images, characters, etc. [6,8]. Research suggests that animated spokes-characters may succeed by creating an emotional connection with the audience through anthropomorphism [7,9]. However, the choice of whether to use a real human or an animated spokesperson to match the informational content of health announcements is very arbitrary, and further exploration is required to understand the interaction between message type (negative versus positive emotion) and spokesperson type (real versus animated) on audience response to messages for pandemic prevention. In this study, the public health ads launched by governments during COVID-19 are tested and further modified to generate advertising stimuli.

Furthermore, consider people also exposed to the ad that read “To protect yourself, you should get vaccinated for COVID-19 as soon as possible” compared to the ad “To protect your family and community, you should get vaccinated for COVID-19 as soon as possible”. The former appeal highlights the benefit to people of getting a COVID-19 vaccine for themselves; in contrast, the latter appeal highlights the benefit for others. Following White [10] and Ryoo [11], we define the former as a “self-benefit” appeal and refer to an appeal emphasizing that the primary beneficiaries of vaccination are those who are vaccinated. We define the latter as an “other-benefit” appeal and, as such, refer to an appeal indicating that the primary beneficiaries of vaccination are other people. Health communicators use benefit appeals in messages (self-benefit or other-benefit) to persuade people to accept a communicator’s recommendation [12,13,14]. Interestingly, despite their extensive use in mass media health campaigns, the interplay between these persuasive strategies has not been jointly examined. There is a need to uncover when one type of appeal is more effective than the other when discussing them in conjunction with the previous two elements of persuasion. Accordingly, the purpose of this study is to empirically examine how different combinations of these persuasive elements in public campaigns affect people’s responses and preventive behaviors in the context of an infectious disease pandemic.

In response, this study first examines how the types of emotional appeals (fear versus efficacy) and spokesperson (real versus animated) interact to influence participants’ attitudes and intention to adopt preventive measures. Furthermore, we find a three-way interaction in which benefit appeals (self-benefit versus other-benefit) moderate the interplay between emotion and spokesperson types on the public’s preventive behaviours. Taken together, the findings of this study contribute to health communication research by examining the appropriate matching between these persuasive variables. In doing so, it enhances our communication skills to increase public acceptance of disease prevention measures.

## 2. Theoretical Background

Improving public vaccination is increasingly becoming an important task for health communicators. Prior research on how fear-arousing messages affect persuasion has made important contributions to the applications of health psychology in public communications.

### 2.1. Emotion Appeals in Health Campaigns

Although the relationship between emotion and behavioral change is not straightforward [2], widespread and rapid promotion of public precautions is not possible without information that includes emotional appeals. Many studies have revealed that emotional engagement can drive people to change their behavior and prior habits [2,3], so emotional appeals are widely used in public health communications [4,5,12]. On the one hand, appeals using nonthreatening language can generate positive emotions and thus increase public receptivity to persuasive messages [12,15,16,17,18]. Positive appeals such as joy, hope, and efficacy can evoke positive emotions [19,20,21,22] and encourage people to act in accordance with public health recommendations. For example, Ojala [20] identified that constructive hope has a significantly positive impact on engagement in pro-environmental activities.

However, other scholars state that negative appeals (e.g., worry, fear) that highlight potential risks might lead to greater persuasiveness by increasing more careful information processing. Robberson [3] pointed out that negative appeals about health hazards were superior to positive appeals about health promotion when persuading people to adopt a healthy lifestyle. Because fear enhances attention to messages due to perceived threat, a fear appeal is more effective at influencing the attitude and behavior of message recipients [4,12]. However, studies have found that overstimulation of negative cognition leads to psychological reactance when persuasive information threatens people’s freedom to choose their behavior [23,24,25]. When people believe they are facing imminent physical or social harm, fear is considered a useful driving force because the associated propensity is to act to protect themselves from harm [3,22,26]. Fear appeals address the severity and salience of a threat to impact behavior [2,4,21]. However, many studies have also found that fear can generate maladaptive information responses, leading to psychological reactance, which may undermine the persuasion process [13,23,24,25].

In research on positive emotions, efficacy perceptions are considered to be constructive responses to threatening situations. In health campaigns, efficacy appeals emphasize that individuals can take successful actions to mitigate the threat of disease or claim that powerful politicians will adopt effective pandemic response programs. Recently, scholars that studied the persuasive influence of efficacy appeals [19,22] found that efficacy messages have the potential to evoke an emotion, i.e., hope, which is critical if calling for more actions when people face a difficult state [22]. Hope is associated with an uncertain future, but it provides more positive expectations of ultimate success. Threatening information usually arouses fear, while efficacy messages tend to evoke hope.

### 2.2. Animated and Human Spokespersons

Within the field of advertising, the use of spokespersons is an important creative advertising strategy [1,7,27]. However, in health communications, a few studies have shed light on the effect of spokespersons. Prior research has indicated that the type of spokesperson can influence behavioral outcomes. Research by Heiser [28] examined consumer responses to animated or cartoon spokespersons in print ads, and identified that using an animated spokesperson in the ad resulted in more positive consumer attitudes and purchase intentions compared to using a human spokesperson in the same print ad. The animated spokesperson is regarded as a creative appeal that can attract more viewers’ attention, and thus enhance persuasiveness. However, other studies have reported different findings. Bhutada [6] conducted experiments to investigate the interactive effect between spokes-character type (human/animated) and level of involvement (low/high). Their findings revealed that participants significantly differed in attitudes towards spokes-characters in the two experimental ad stimuli (more positive attitudes towards the real human spokesperson). In some studies focusing on interaction effects, the boundary conditions for animated and human spokes-characters were examined. A study by Newton [29] found that using an anthropomorphized digestive system as a spokes-character in threatening health messages reduced participants’ portion preference for energy-dense foods and drinks. However, this effect is only significant for those who have a low sense of power because those types of people are more sensitive to the social influences conveyed by organ anthropomorphism and more receptive to suggestions of threatening health information. We find that the effect of spokesperson type is inconsistent in different research contexts.

An animated spokesperson is actually an image design with a human as a reference object [9,27,30,31]. On the one hand, when people see animated characters in ads, they can clearly perceive the difference between an animated spokesperson and a real human, although there are many similarities between the two types. These similarities can evoke consumers’ perception of schema, such as interpersonal influence. Research on anthropomorphism often finds that people respond to anthropomorphized objects similarly to how they respond to humans in the same contexts [9,27]. On the other hand, animated spokespersons make people feel more relaxed and happier [32,33] because they enable consumers to accept persuasive messages with a sense of freedom [31] compared with the persuasive message conveyed by a real person, which leads to perceived control, social norms, and social pressure [28].

## 3. Hypothesis Development

### 3.1. Emotion Appeals and Spokespersons

Our knowledge on the effect of animated spokespersons in health campaigns is still deficient, and little research has investigated the interactive effect of message type and spokesperson type. The purpose of this study is to expand our understanding of how the interplay between emotional appeals (fear versus efficacy messages) and spokesperson type (real people versus animated characters) affects viewers’ response to public campaigns for the prevention of pandemics.

The first aim of this study is to compare fear appeals with their corresponding efficacy appeals. Efficacy appeals emphasize the effectiveness of given solutions and achieve positive results [19]. Efficacy appeals encourage people to pursue goals and relieve them from negative situations. However, fear appeals depict the consequences of not following the communicator’s advice, resulting in bodily harm or even death [2,3,21]. When a communicator is trying to convince people to engage in a certain behavior for their health, a threatening message is probably more persuasive than a positive message [2,3,4,12]. Negative messages can enhance people’s perceived severity of health threats more than positive messages. Obviously, the allure of gaining the benefits of health protection is not as persuasive as avoiding the negative consequences of health loss. Thus, we propose that the fear message would trigger higher perceived severity than the efficacy message. Furthermore, although the audience finds it more difficult to accept fear messages psychologically than efficacy messages, we believe that fear messages can lead to a higher intention to take preventive actions.

The second goal of this study is to compare real and animated spokespersons and examine how they interact with emotional appeals to influence the persuasiveness of communicators’ recommendations. The animation spokesperson is an application of an anthropomorphic communication strategy. Anthropomorphism involves imbuing nonhuman entities with human features such as giving a humanlike appearance or the ability to think or to talk [27,28,29,30]. Anthropomorphism enables humans to quickly judge whether something is threatening [9,27,30]. The research of Chandler [34] indicates that after anthropomorphizing objects, people think about the object from a subjective perspective, such as feeling the emotional warmth that the object brings. Previous studies have also shown that in the cognitive processing of anthropomorphism, people experience perceptual fluency, that is, the subjective sense of ease and pleasure in information processing [9,29,32,33]. The use of animated spokes-characters may reduce viewers’ pressure on social norms in interpersonal interactions precisely because animated spokes-characters are not real humans [29,30]. When viewers read the suggestions and the given solutions proposed by animated spokespersons, viewers feel a more relaxed mental state compared with reading the suggestions made by a real human. We suggest that viewers’ responses to message appeals may be jointly impacted by the types of spokesperson featured in the ads. Specifically, we propose that in public ads, efficacy messages combined with animated spokespersons will result in audiences’ lower perceived severity and better public acceptance. However, the reduction in the intensity of information threats may lead to a lower intention to take protective actions.

Evoking emotion (fear or efficacy) can potently increase people’s attention and motivation to process health messages [2,15,19,21,22]. However, we expect that the spokesperson may have an interaction effect with emotional appeals. Fear-arousing messages traditionally depict the negative, aversive consequences if not doing what the communicator recommends [3]. Pairing a human spokesperson with a fear message can better emphasize the message by providing social pressure. In addition, matching an animated spokesperson with an efficacy message will result in lower risk perception. Thus, we propose the following:

**H1:**  *Individuals who read a fear message in the epidemic prevention poster will report higher perceived severity when exposed to a real spokesperson (versus an animated spokesperson).*

**H2:**  *Individuals who read an efficacy message in the epidemic prevention poster will report higher message receptivity when exposed to an animated spokesperson (versus a real spokesperson).*

### 3.2. Self-Benefit Appeal versus Other-Benefit Appeal

Another strategy that health promoters often consider is “benefit-target framing”, which proves useful in promoting vaccine intentions [12,35,36]. The literature has classified benefit appeals into two types according to the beneficiaries of the actions [10,11,14,36,37]. Self-benefit appeals highlight the self as the primary beneficiary of the action, while other-benefit appeals emphasize that other individuals are the primary beneficiaries of the action. The third aim of this study is to investigate whether a message promoting an individual being vaccinated against epidemic diseases for the self (by using self-benefit appeals) is more persuasive than that for loved ones and unknown others in the community (by using other-benefit appeals) or when one type of benefit appeal is better than another in encouraging vaccination and other prevention decisions.

The theory of protection motivation assumes that people have motivation to protect themselves from physical danger, social danger, and psychological danger [3]. Some studies suggest that self-benefit appeals are particularly effective in cognitive and behavioral modifications such as increased willingness to donate [11,38] because favorable cost-benefit ratios are highlighted. Self-benefit appeals focus on the benefits to individuals after engaging in the suggested behavior, which makes a clear and direct conviction. In contrast, White [10] found that other-benefit appeals generated more positive responses than self-benefit appeals when public self-image was emphasized. That is, people can engage in disease-preventive behavior for reasons other than protecting their own health. In a study of avian influenza vaccination intentions, Ceylan [12] also demonstrated that messages emphasizing the social benefits of vaccination were more effective than self-interested framing messages. Self-benefit appeals in health campaigns mainly contribute to fulfilling the target audience’s self-interested motives, while other-benefit appeals help to achieve altruistic motivations.

In different research contexts, the conclusions of previous studies are not consistent. Moreover, prior studies compare only the persuasive effect of different types of benefit appeals. However, in real public health campaigns, the posters contain multiple persuasive elements. In this study, we combine benefit appeals with the previously discussed emotion appeals and spokesperson types for multiple comparisons. The research findings will have more ecological validity and practical application significance.

As we predicted before, when matched with a real spokesperson (versus an animated spokesperson), fear messages lead to higher perceived severity because the real spokesperson provides a real example of a warning signal for message processing, which leads to higher intentions to take preventive actions. The above hypotheses combined with self-benefit appeals can emphasize the serious consequences of losing personal health, thus forming a more consistent persuasive message. Adopting disease-prevention behavior is virtually the only way to achieve the goals of personal health described in the health message (e.g., wearing a face mask outside, cleaning hands often, limiting outdoor activities, getting vaccinated/receiving the vaccine).

In contrast, when other-benefit appeals are used, it is more suitable to match efficacy messages and animated spokespersons. The prevention and treatment of infectious diseases cannot be completely avoided by individual protection but requires the joint efforts of the whole society. However, people cannot take responsibility for the actions of others, which results in feelings of powerlessness. Using efficacy messages matched with animated spokespersons makes people feel relaxed and improves the public’s confidence and hope, so that they have higher message receptivity and are confident in working together and participating in prevention behavior (e.g., keeping social distance from others, avoiding mass gatherings, using mobile health advice apps). Therefore, we propose the following hypothesis:

**H3:**  *Benefit appeals will moderate the interaction between emotional appeals and spokesperson types. Specifically, individuals’ preventive intentions will be greater with (a) a self-benefit appeal for the match between a fear message and a real spokesperson (compared to an other-benefit appeal) and (b) an other-benefit appeal for the match between an efficacy message and an animated spokesperson (compared to a self-benefit appeal).*

## 4. Methods

### 4.1. Experimental Procedure

We first conducted a pilot experimental study to determine whether the treatments were effective. Second, we conducted a main experiment to examine the interplay of emotional appeals and spokesperson types in the context of providing guidance to communities facing the challenge of the pandemic to engage in preventive behavior. Third, we examined whether benefit appeals moderate the relationship between message type and spokesperson type regarding intention to adopt preventive behavior.

### 4.2. Study Design

The most appropriate method to examine the hypotheses is framing experiments, in which information about adopting disease-preventive behavior might be framed as emphasizing either self- or other-benefits and framed as focusing on health threats or efficacy. A 2 (emotional appeal: fear versus efficacy) × 2 (spokesperson type: human versus animated) × 2 (benefit appeal: self-benefit versus other-benefit) between-subjects experimental design was employed. We browsed all the ads released by the Macao government during the COVID-19 pandemic and selected two public health announcements as original ad stimuli. Then, other versions of comparison stimuli were created by revising the two original ads (see Appendix A). To avoid any bias effects caused by image differences, we used the same color and background image in each stimulus across the eight experimental groups.

Message type was manipulated by the text in the ad stimuli. The first ad stated text triggering fear as a negative emotion (“Elderly people are more susceptible to infection, and they are also prone to severe illness or even death after infection”, “At present, the vaccination rate for the elderly in Macao is seriously low”), while the second ad delivered text with confidence and efficacy as a positive emotion (“The confirmed patients recovered and were discharged from hospital, strengthening Macao’s confidence in fighting against the epidemic”, “The government has the confidence to do a good job in the fight against the epidemic, and hope that the public can rest assured”). Consistent with a previous study [4], the non-animated ad copy depicted a real human spokesperson in the ads, while the animated version contained an animated spokesperson in the ads. Benefit appeals were primed through the ad text. In the self-benefit condition, the text addressed the self-benefit of getting a COVID-19 vaccine (“To protect yourself, you should receive the new coronavirus vaccine as soon as possible”). In the other-benefit condition, the text emphasized getting a COVID-19 vaccine to protect others (“To protect your family and community, you should receive the new coronavirus vaccine as soon as possible”).

### 4.3. Pilot Study: Experimental Stimuli Development

The purpose of the pilot study was to check whether the treatments successfully produce the intended response [39]. A total of 149 participants (83 women) from a large university in Macao participated in the study in exchange for course credit. After consenting to the study, the participants were randomly assigned to one of eight experimental conditions. After seeing the ad stimuli, the participants indicated their perception of the ads. We removed 2 participants for incomplete data, and 147 valid responses were used (Mage = 19.7, SD = 0.97, 56.5% female).

The manipulation of fear messages was assessed by a 7-point item: “The ad I viewed focused on the worst consequences of the epidemic”. The manipulation of efficacy messages was assessed by a 7-point item “The ad I viewed focused on confidence in good outcomes for treating the epidemic” (1 = least likely and 7 = most likely). Adopted from a prior study [27], we asked participants to indicate the source of the message using a 7-point item “Who delivered the message?” (1 = an animated source and 7 = a human source). The manipulation of self-benefit appeal was assessed by a 7-point item “The ad I viewed mainly emphasized the benefit of vaccine for my health”, and the manipulation of other-benefit appeal was assessed by a 7-point item “The ad I viewed mainly emphasized the benefit of vaccine for others” (1 = least likely and 7 = most likely).

To assess the manipulation of emotional appeal, a 2 (emotional appeals: fear versus efficacy) × 2 (spokesperson type: real versus animated) × 2 (benefit appeals: self-benefit versus other-benefit) three-way between-subjects ANOVA was conducted using the questions of the manipulation check as the dependent variable. As we predicted, participants who read the fear message indicated a higher score for “The ad I viewed focused on the worst consequences of the epidemic” (M = 5.28, SD = 1.19) than those who read the efficacy message (M = 2.71, SD = 1.02), and the main effect of emotional appeals was significant, F(1,139) = 196.32, *p* < 0.001, while no other main effects were significant. However, participants who read the efficacy message indicated higher scores for “The ad I viewed focused on confidence in good outcomes for treating the epidemic” (M = 5.32, SD = 1.09) than those who read the fear message (M = 2.92, SD = 1.00), and the main effect of emotional appeals was significant, F(1,139) = 187.90, *p* < 0.001, while no other main effects were significant.

To test the manipulation of spokesperson type, a three-way between-subject ANOVA was conducted using the item “Who delivered the message?” (1 = an animated source and 7 = a human source) as the dependent variable. As we expected, participants who were exposed to the message with a human indicated higher scores (M = 5.98, SD = 0.85) than those exposed to the animated spokesperson (M = 2.47, SD = 1.00), and the main effect of spokesperson type was significant, F(1,139) = 542.62, *p* < 0.001, while no other main effects were significant.

To assess the manipulation of benefit appeals, a three-way between-subject ANOVA was conducted using the question of manipulation check as the dependent variable. As we predicted, participants who read the message highlighting the self-benefit of getting a vaccine indicated a higher score of “The ad I viewed mainly emphasized the benefit of vaccine for my health” (M = 5.66, SD = 1.00) than those who read the other-benefit appeal (M = 2.01, SD = 0.88), and the main effect of benefit appeals was significant, F(1,139) = 552.69, *p* < 0.001, while no other main effects were significant. However, participants who read the message emphasizing others’ benefit indicated higher scores for “The ad I viewed mainly emphasized the benefit of vaccine for others’ health” (M = 5.67, SD = 1.00) than those who read the self-benefit appeal (M = 2.12, SD = 0.96), and the main effect of benefit appeal was significant, F(1,139) = 477.35, *p* < 0.001, while no other main effects were significant. Thus, the results indicated that all manipulations of the independent variables were effective and that the eight ads could be used in the following main study.

### 4.4. Main Experiment: Hypothesis Test

We collected the data in November 2021. Although the new coronavirus epidemic had lasted for more than a year, due to the continuous mutation of the virus, Macao was still in the stage of strict prevention and control. A total of 1117 Macao residents were recruited on Sojump (an online crowdsourcing platform providing functions equivalent to Amazon Mechanical Turk). After consenting to the study, the participants were randomly assigned to one of eight experimental conditions. All other test procedures were controlled to be consistent across the eight groups to avoid any confounding effects. Thus, any difference in attitude evaluation of the ads in this context would be related to the differences between the stimuli and not to a preference for other factors. The participants took one more minute to view the ad stimuli. Then, we measured persuasion by assessing participants’ attitudes and behavioral intentions regarding epidemic control and prevention. Specifically, we measured their (1) perceived severity, (2) message receptivity, (3) intentions to take preventive actions, and (4) demographic information. After removing 22 participants for incomplete data, 1095 valid responses were used.

### 4.5. Measures

Regarding our primary variable of interest, perceived severity was a 7-point, three-item scale adopted from Luo [40], including (1) I believe that the pandemic is a deadly disease; (2) I believe that the pandemic can bring severe health problems; and (3) I believe that the pandemic is a serious threat to my health. We measured message receptivity using a 7-point, three-item scale adopted from Ilakkuvan [41], including (1) This ad grabbed my attention; (2) This ad was worth remembering; and (3) This ad was convincing (1 = strongly disagree and 7 = strongly agree). Next, we measured participants’ intentions to take preventive actions by asking them the degree to which they like to (1) clean hands often, (2) wear a face mask outside, (3) receive the vaccine, and (4) keep social distance from others (1 = least likely and 7 = most likely), adopted from Luo [40] and OECD [42].

### 4.6. Results

#### 4.6.1. Demographic Characteristics

Our respondents consisted of 47.1% men (*n* = 516) and 52.9% women (*n* = 579). Regarding age distribution, 20.6% of respondents (*n* = 226) were 55 years old and above, 24.5% of respondents (*n* = 268) were 40–55 years old, 39.2% of respondents (*n* = 429) were 25–39 years old, and 15.7% of respondents (*n* = 172) were 18–24 years old. Among them, more than three-quarters of the respondents (*n* = 883) reported obtaining an associate’s degree or above. Chi-squared tests confirmed that there were no significant differences between the eight experimental groups in terms of demographic characteristics. An overview of the respondents’ demographic characteristics is shown in Table 1.

#### 4.6.2. Hypothesis Testing

The items of perceived severity demonstrated good internal consistency (reliability a = 0.927). Thus, we generated a composite score, averaging the items. Other constructs were averaged since reliability alphas were all in the acceptable range: message receptivity (a = 0.890) and intentions to take preventive actions (a = 0.931).

Hypothesis 1 proposed that individuals reading a fear message in the epidemic prevention poster will report higher perceived severity when exposed to a real spokesperson (versus an animated spokesperson). To examine those predictions, a 2 (emotional appeal: fear versus efficacy messages) × 2 (spokesperson type: human versus animated) between-subjects ANOVA was conducted using perceived severity as the dependent variable. The results demonstrated a significant two-way interaction between emotional appeal and spokesperson type, F(1,1091) = 10.774, *p* < 0.01, partial *η*^2^ = 0.01 (see Table 2).

As predicted, participants read a fear message illustrated by a real spokesperson that indicated a higher level of perceived severity (M = 5.731, SD = 0.731) compared to the message illustrated by an animated spokesperson (M = 5.081, SD = 0.723); F(1,1091) = 56.486, *p* < 0.001. Similarly, participants who read an efficacy message illustrated by an animated spokesperson (M = 2.522, SD = 1.025) reported a lower level of perceived severity than those who read an efficacy message illustrated by a real spokesperson (M = 3.574, SD = 1.414); F(1,1091) = 147.156, *p* < 0.001. Thus, H1 is supported.

On the other hand, Hypothesis 2 proposed that individuals reading an efficacy message in the epidemic prevention poster will report higher message receptivity when exposed to an animated spokesperson (versus a real spokesperson). Then, a 2 × 2 (emotional appeal by spokesperson type) ANOVA was conducted on participant ratings of message receptivity. This analysis indicated that there was a significant interaction effect between emotional appeal and spokesperson type, F(1,1091) = 8.065, *p* < 0.01, partial *η*^2^ = 0.007 (see Table 2). As predicted, participants reading a fear message illustrated by a real spokesperson indicated a lower level of message receptivity (M = 3.442, SD = 1.677) compared to the message illustrated by an animated spokesperson (M = 4.067, SD = 0.916); F(1,1091) = 37.585, *p* < 0.001. Similarly, participants who read an efficacy message illustrated by an animated spokesperson (M = 5.156, SD = 1.039) reported a higher level of message receptivity than those who read an efficacy message illustrated by a real spokesperson (M = 4.120, SD = 0.981); F(1,1091) = 102.508, *p* < 0.001. Thus, H2 is supported.

Hypothesis 3 predicted that benefit appeal moderates the interplay between emotional appeal and spokesperson type. A 2 × 2 × 2 (emotional appeal by spokesperson type by benefit appeal) ANOVA was conducted with individuals’ intentions to take preventive actions as the dependent measure. This analysis indicated that there was significant two-way interaction effect between emotional appeal and benefit appeal, F(1,1087) = 984.706, *p* < 0.001. The two-way interaction effect between spokesperson type and benefit appeal was significant, F(1,1087) = 6.253, *p* < 0.05. More importantly, the three-way interaction effect between emotional appeal, spokesperson type, and benefit appeal was significant, F(1,1087) = 4.858, *p* < 0.05 (see Table 3).

As shown in Table 4, the simple main effect test also revealed that when participants read a fear message illustrated by a real spokesperson, if the public announcement emphasized getting a vaccine against epidemic diseases for their own health (self-benefit appeals), it yielded greater preventive intention (M = 5.583, SD = 1.259) than emphasizing the health of loved ones and unknown community members (other-benefit appeals) (M = 2.882, SD = 1.414), F(1,1087) = 350.597, *p* < 0.001 (see Table 4).

In contrast, when participants read an efficacy message illustrated by an animated spokesperson, if the public announcement highlights taking preventive actions for the health of loved ones and unknown community members (other-benefit appeals), it led to greater preventive intention (M = 5.532, SD = 0.980) than highlighting their own health (self-benefit appeals) (M = 3.325, SD = 1.310), F(1,1087) = 230.568, *p* < 0.001. Thus, H3 is supported.

## 5. Discussion

### 5.1. Theoretical and Practical Implications

Epidemic outbreaks have had a significant negative impact on normal economic and social activities [43,44]. Evidence suggested that public prevention campaigns could be a powerful motivator for adopting preventive measures [45,46]. Therefore, public health officials need strategies for public prevention and vaccine promotion. The first aim of this study was to examine how different combinations of emotional appeal and spokesperson type in health campaigns affect people’s perceived severity and message receptivity in the context of a pandemic. Spokes-characters have been used as a significant persuasive visual tool in advertising [1,7,28], but few prior studies on public health ads have investigated the effectiveness of real humans or animated spokespersons in matching the informational content in health announcements [6,29]. Fear-arousing messages depicted the negative, aversive consequences if what the communicator recommends was not done [3]. As we predicted, when viewers read the fear message proposed by real human spokespersons, viewers felt more realistic and perceived severity, which encouraged them to act in accordance with health recommendations. In contrast, efficacy perceptions were considered to be constructive responses to threatening situations [15,19,22]. Compared with the efficacy message conveyed by a real person, animated spokespersons made people feel more relaxed and happier [30,32,33]. When an efficacy message was illustrated by animated spokespersons, it enhanced public acceptance of disease prevention recommendations. These research findings have important implications for health communication researchers and practitioners.

Furthermore, our results suggested that benefit appeals moderated the interaction between emotional appeals and spokesperson types. The joint effect of these persuasive variables on individual’s intention to take preventive actions indicated that the interactions (emotional appeal × benefit appeal, spokesperson type × benefit appeal, and emotional appeal × spokesperson type × benefit appeal) significantly differed across the two types of benefit appeal. Prior studies that assessed the health message framing during the COVID-19 pandemic have not examined the relationship between these persuasive strategies nor have they identified the most effective combination of these persuasive elements to persuade the public to engage in prevention behavior [45,46]. We predicted when people perceived health threats, fear was considered a useful driving force to protect themselves from harm [3,22], while self-benefit appeals focused on the personal benefits to the individuals after taking the suggested preventive behavior [11,38,43]. Matching this type of health message with a real human spokesperson resulted in a clear and direct conviction since the real spokesperson provided a real example of a warning signal for the prevention message. On the other hand, the prevention and treatment of infectious diseases could not rely solely on the preventive behavior of individuals but required the joint efforts of society as a whole [44,47,48]. However, people cannot take responsibility for the actions of others [49,50]. Fear messages only increased the public’s sense of powerlessness [48], whereas the adoption of efficacy messages could evoke hope, which was crucial in calling for positive action in the face of adversity [22]. Other-benefit appeals help to achieve this altruistic motivation [49,50]. Altruistic motives, such as efficacy messages, brought about positive emotions such as joy and hope when faced with difficult situations. Therefore, using efficacy messages matched with other-benefit appeals raised the public’s confidence, leading to higher acceptance of prevention recommendations. As we predicted, the results indicated that the match between a fear message and a real human spokesperson elicited the greatest individuals’ preventive intentions with a self-benefit appeal, whereas the match effect between an efficacy message and an animated spokesperson elicited the greatest individuals’ preventive intentions with an other-benefit appeal.

In conclusion, our findings provide valuable insights and recommendations for health communicators seeking to enhance public communication strategies. Firstly, it is crucial for health advertising to emphasize that adopting preventive measures not only safeguards oneself but also protects others. By highlighting collective responsibility, individuals are more likely to perceive the importance of their actions in safeguarding public health. Moreover, tailoring the message to evoke the appropriate emotional response is essential. Fear messages are effective in motivating self-protection, while efficacy messages promote willingness to protect others and are more likely to be accepted by the public. In addition, the choice of spokespersons could significantly impact the message’s reception. A real spokesperson enhances the perceived danger when delivering a fear message, while a cartoon spokesperson improves public acceptance when advocating for protecting others. By integrating these findings into health advertising campaigns, the overall effectiveness of public communication can be enhanced. Consequently, the public’s acceptance of disease prevention measures and their willingness to take timely action can be significantly improved.

### 5.2. Limitations and Future Research

The findings of this research are subject to a few limitations. First, the findings of this study are based on cross-sectional data collection. In real life, the public’s behavioral response to prevention advice issued by the government is constantly changing as information about epidemics continues to be exposed [44,47,49]. Research on the effectiveness of persuasive elements in health announcements requires follow-up studies at different points in time. Another limitation of this research is that the data collected during the new coronavirus epidemic may have amplified the impact of the health posters in the experiment on the subjects because the government had already raised public awareness about the prevention of epidemics through a great deal of publicity during this period [45,47,50]. Would the public have responded the same way in other disease prevention or health campaigns when they saw several of the persuasive strategies examined in this study? Clearly, more research is needed before any generalizations can be made.

Our findings are also limited by the fact that we did not exclude some confounding variables, such as the possibility that the subjects themselves or their family members might have been infected with the new coronavirus epidemic before participating in the experiment. This would have increased their perceived severity of the infection and their willingness to take strict preventive measures. More confounding variables need to be measured and controlled for in subsequent studies. Finally, there is a need to explore the underlying psychological mechanisms that may explain why the match between emotional appeal and spokesperson type, and the match between emotional appeal and benefit appeal, lead to greater preventive intentions. Thus, future research should identify the potential mediators to better understand the combination of persuasive strategies. Despite these limitations, this study provides valuable implications for theoretical and practical health communications to help flatten the epidemic curve by examining the combined effects of these persuasive strategies.

## Figures and Tables

**Table 1:** Demographic characteristics of respondents for the study (*n* = 1095).

Variable	Categories	N	Percentage (%)
Gender	Male	516	47.1%
	Female	579	52.9%
Age	18–24	172	15.7%
	25–39	429	39.2%
	40–54	268	24.5%
	55 and above	226	20.6%
Education	High school diploma or less	262	23.9%
	Associate’s degree	295	26.9%
	Bachelor’s degree	334	30.5%
	Above Bachelor	204	18.6%

**Table 2:** Univariate analyses on perceived severity and message receptivity.

Source	Dependent Variable	Mean Square	F	*p* Value	Partial *η*^2^
Emotional Appeal (EA)	Perceived Severity	1521.401	1481.627	<0.001	0.576
Spokesperson Type (ST)		198.300	193.116	<0.001	0.150
EA × ST		11.063	10.774	0.001	0.010
*R*^2^ = 0.607 (Adjusted *R*^2^ = 0.606)					
Emotional Appeal (EA)	Message Receptivity	213.649	149.721	<0.001	0.121
Spokesperson Type (EA)		188.655	132.206	<0.001	0.108
EA × ST		11.508	8.065	0.005	0.007
*R*^2^ = 0.210 (Adjusted *R*^2^ = 0.208)					

**Table 3:** Univariate analyses on preventive intentions.

Source	Mean Square	F	*p* Value	Partial *η*^2^	Observed Power
Emotional Appeal (EA)	6.342	4.416	0.036 *	0.004	0.556
Spokesperson Type (ST)	0.518	0.361	0.548	0.000	0.092
Benefit Appeal (BA)	2.116	1.473	0.225	0.001	0.228
EA × ST	2.992	2.084	0.149	0.002	0.303
EA × BA	1414.052	984.706	<0.001 ***	0.475	1.000
ST × BA	8.979	6.253	0.013 *	0.006	0.705
EA × ST × BA	6.977	4.858	0.028 *	0.004	0.596

Note: Dependent variable: individuals’ intentions to take preventive actions; *R*^2^ = 0.480 (Adjusted *R*^2^ = 0.477); * *p* < 0.05; *** *p* < 0.001.

**Table 4:** Pairwise comparisons.

		Self-Benefit	Other-Benefit	Mean Square	F	*p* Value
**Fear**	**Real**	Mean = 5.583	Mean = 2.882	503.462	350.597	<0.001
		SD = 1.259	SD = 1.414			
		(*n* = 141)	(*n* = 135)			
	**Animated**	Mean = 5.182	Mean = 3.161	278.522	193.955	<0.001
		SD = 1.228	SD = 1.022			
		(*n* = 135)	(*n* = 138)			
**Efficacy**	**Real**	Mean = 3.198	Mean = 5.362	320.543	223.217	<0.001
		SD = 1.147	SD = 1.166			
		(*n* = 140)	(*n* = 134)			
	**Animated**	Mean = 3.325	Mean = 5.532	331.099	230.568	<0.001
		SD = 1.310	SD = 0.980			
		(*n* = 137)	(*n* = 135)			

Note: Dependent variable: individual’s’ intentions to take preventive actions.

**Figure A1:**
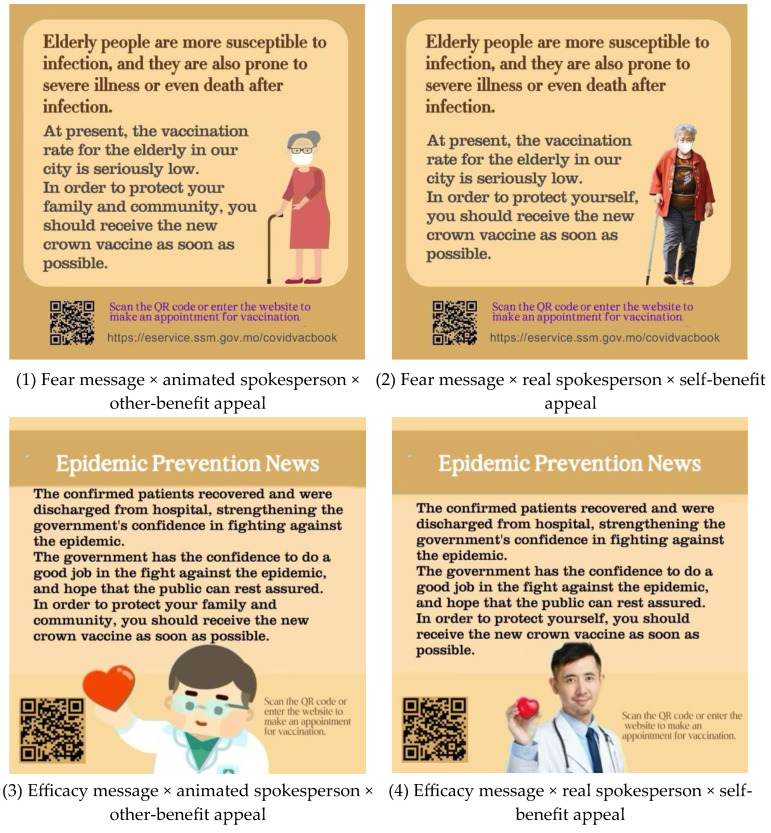
Advertising stimuli used in this study.

## Data Availability

The data presented in this study are available on request from the corresponding author.
